# Cooperation between monocytes and breast cancer cells promotes factors involved in cancer aggressiveness

**DOI:** 10.1038/sj.bjc.6600872

**Published:** 2003-04-15

**Authors:** E Blot, W Chen, M Vasse, J Paysant, C Denoyelle, J-Y Pillé, L Vincent, J-P Vannier, J Soria, C Soria

**Affiliations:** 1 DIFEMA Laboratory, Medicine and Pharmacy Faculty, 22 boulevard Gambetta, 76183 Rouen, cedex, France; 2 INSERM EMI 9912 and Biochemistry and Sainte Marie Laboratories, Hôtel-Dieu, place du Parvis de Notre-Dame, 75004 Paris, France; 3 Départment d'Oncologie Médicale, Henri Becquerel Center, rue d'Amiens, 76038 Rouen, cedex1, France; 4 INSERM U353, Hôpital St Louis, avenue Claude Vellefaux, 75010 Paris, France

**Keywords:** breast cancer, monocytes, inflammation, cytokine, invasiveness factors

## Abstract

In breast cancers, clinical symptoms of inflammation localised around the tumour at the time of diagnosis have been considered to have poor prognosis significance. In this study, the biological mechanisms responsible for the deleterious action of monocytes in cancer were investigated. The incubation of the breast-cancer-derived MDA-MB231 cells with monocytes resulted in an increase in factors involved in cell invasion (i.e. both cancer cells and monocytes-associated urokinase and Tissue Factor, and PAI-1 and MMP-9 secretion). Moreover, the functions of monocytes were also modified. Incubation of monocytes with MDA-MB231 cancer cells resulted in a downregulation in the secretion of the antiproliferative cytokine Oncostatin M, while the apoptotic factor TNF alpha was dramatically increased. However, MDA-MB231 cancer cells have been shown to be resistant towards the apoptotic action of TNF alpha. These findings demonstrate that incubation of MDA-MB231 cancer cells with monocytes induced a crosstalk, which resulted in an increased expression of factors involved in cancer cell invasiveness and in a modification of monocytes function against cancer cells, while inflammatory effects were increased.

The importance of stroma in cancer formation, growth and dissemination is now well established. In order to generate an invasive tumour, cancer cells need several mutations, but also formally necessitate sustenance from noncancer stroma cells, which supply and permit cancer cell growth and invasion. The presence of cytokines and inflammatory cells in cancer stroma, indicating defence reaction against cancer, generally correspond to a poor prognosis (Kleer *et al*, 2000). Furthermore, in breast cancer, initial presentation with clinical symptoms of inflammation indicates a particular aggressiveness and a worst prognosis than other breast cancers (Chevallier, 1993; Palangie *et al*, 1994; Kleer *et al*, 2000). However, the mechanisms leading to the pathological action of stroma-localised inflammatory cells in cancer, such as monocytes, remain poorly understood.

The current study was undertaken to explore whether the factors of aggressiveness expressed by cancer cells were modified in the presence of monocytes. We also analysed the consequences of the incubation of monocytes with MDA-MB231 cancer cells on functions of monocytes both in the defence against tumour cells and in inflammatory functions. For this purpose, we tested the consequences of the interaction of the highly invasive breast-derived MDA-MB231 cancer cells with monocytes with an *in vitro* system.

## MATERIAL AND METHODS

### Monocytes isolation

A measure of 80 mm of blood from healthy volunteers was collected in sterile polypropylene tubes (Costar, Cambridge, MA, USA) containing 7.5 ml of ACD (38 mM citric acid, 74 mM citrate and 136 mM glucose). Platelet-rich plasma was removed by centrifugation (10 min, 100 **g** at room temperature). Mononuclear cells were collected after centrifugation (45 min, 400 **g** at room temperature) in Leucosep tubes (Costar, Cambridge, MA, USA) containing 15 ml of Ficoll (Diatrizoate-Ficoll *d*=1.077, Eurobio, Les Ullis, France). Mononuclear cells were then washed twice with RPMI (RPMI 1640, Gibco, New York, NY, USA). These cells were then resuspended and incubated in 10 ml of RPMI with 10% foetal calf serum (FCS) (Gibco, New York, NY, USA) 1% glutamine, 100 U ml^−1^ penicillin (Sarbach, Suresnes, France) and 100 *μ*g ml^−1^ streptomycin (Diamant, Puteaux, France) in 75 ml flasks for 45 min (5% CO_2_, 95% water-saturated air mixture at 37°C). Nonadherent cells were removed. Adherent cells (approximately 90% monocytes) were then washed twice with RPMI, scrapped, washed and adjusted at 10^6^ ml^−1^ in RPMI with 10% FCS, 1% glutamine, 100 U ml^−1^ penicillin and 100 *μ*g ml^−1^ streptomycin.

### Cell culture

MDA-MB231 cancer cells were cultured in RPMI containing 10% FCS, 1% glutamine, 100 U ml^−1^ penicillin and 100 *μ*g ml^−1^ streptomycin.

### Incubation of MDA-MB231 cancer cells and monocytes

MDA-MB231 cancer cells were plated in 24-well plates (10^6^ cells by well) for 24 h, to achieve 80% confluence in the wells. Then 0.5 × 10^6^ monocytes were added into the wells and incubated for 24 h at 37°C. Controls consisted of monocytes and MDA-MB231 cancer cells alone.

After incubation, the supernatants were collected for cytokine measurements and cells were collected for evaluation of membrane-associated tissue factor (TF), urokinase (u-PA) and u-PAR by flow cytometry.

### Zymography assays for metalloproteinases (MMPs) and u-PA secretions in supernatants

Supernatants of MDA-MB231 cancer cells cultured for 18 and 48 h, in the absence or presence of monocytes as indicated above, were tested for MMP and u-PA by zymography.

For MMP assessment, supernatants were electrophorised on 7.5% polyacrylamide gel containing 10% SDS and gelatine (1 mg ml^−1^) under nonreducing conditions. After electrophoresis, SDS was removed from the gels by washing for 1 h in 2.5% triton X-100 at room temperature. Gelatinase activity was revealed overnight at 37°C in a buffer containing 50 mM Tris-HCl and 5 mM calcium chloride. The gels were stained with 0.25% Commassie blue R250 (Sigma, Saint Quentin Fallavier, France) and gelatinase activity was observed as clear bands against the blue background of stained gelatine. Molecular weights were determined using prestained standards.

For u-PA secretion, zymography was performed according to Granelli-Piperno and Reich (1978).

### Flow cytometry analysis of u-PA, u-PAR and TF expression on MDA-MB231 cancer cells and monocyte membranes

A phycoerythrin-conjugated (PE) antibody against CD14 (Imunotech, Marseille, France) recognising monocytes cells by direct immunofluorescence was used to make the distinction between cancer cells and monocytes. The cell-membrane-associated u-PA and -TF were determined by indirect immunofluorescence using first a murine monoclonal antibody against human u-PA *β*-chain (American diagnostica, Greenwich, CT, USA) or against TF (Serbio, Genevilliers, France) and secondly a fluorescein isothiocyanate (FITC)-conjugated anti-mouse IgG (Immunotech, Marseille, France). The cell-membrane-associated u-PAR was determined by direct immunofluorescence using phycoerythrin-conjugated antibody against CD87 (Pharmingen, San Diego, CA, USA).

After incubation of MDA-MB231 cancer cells with monocytes, as described above, cells were detached using nonenzymatic detachment solution (Sigma, Saint Quentin Fallavier, France). Then 2 × 10^6^ cells were resuspended and washed twice in phosphate buffered saline (PBS). The first antibody (100 *μ*l at 10 *μ*g ml^−1^) was added to the cells and after a 15-min incubation period (4°C), cells were washed twice with 2 ml of PBS. The second antibody (FITC-labelled anti- mouse antibodies) was then added (100 *μ*l at 10 *μ*g ml^−1^) and cells were further incubated 15 min at 4°C. After washing and centrifugation, the PE-labelled antibody against CD14 was added and the last incubation was performed for 15 min at 4°C. Cells were then washed, centrifuged and resuspended in 1 ml of PBS. Flow cytometry was performed with cytometer EPICS XL-MCL (Coulter, USA) and the mean of fluorescence was measured. On the FS Log *vs* SS Log cytogram, two regions were created corresponding to monocytes and MDA-MB231 cancer cells. Fluorescence histograms were associated with these two regions. For each experiment, the purity of the populations defined on the FS Log *vs* SS Log was analysed by labelling monocytes with a FITC-conjugated antibody against CD14. Less than 5% of cells in MDA-MB231 cancer cell region were positive for CD14, while more than 95% were positive in the monocyte region.

### PAI-1, Oncostatin M and TNF alpha in supernatants

PAI-1, OSM and TNF alpha concentrations were determined in the supernatants of incubation of monocytes with MDA-MB231 cancer cells and in the control supernatants (monocytes and MDA-MB231 cancer cells alone).

The assays were performed by ELISA assays using Quantikine Oncostatin (R&D systems, Abington, UK), Quantikine Elisa TNF alpha kit (R&D systems, Abington, UK) and PAI-1 (Asserachrom PAI-1, Diagnostic Stago, Asnières, France).

### Assessment of TNF alpha-induced apoptosis on MDA-MB231 cancer cells

Assessment was performed by an analysis of cell cycle distribution on MDA-MB231 cancer cells whether or not treated by TNF alpha at 50 ng ml^−1^ for 72 h. The Vindelov technique was used (Vindelov and Christensen, 1990). Briefly, cells were harvested by gentle scraping, pelleted by centrifugation and resuspended in 70 *μ*l of citrate buffer (250 mM sucrose, 40 mM trisodium citrate, 5% DMSO, pH 7.6). Then, cells were treated with 350 *μ*l of trypsin (30 *μ*g ml^−1^) (Sigma, Saint Quentin Fallavier, France) for 10 min at room temperature followed by treatment with 300 *μ*l of trypsin inhibitor (0.5 mg ml^−1^) (Sigma, Saint Quentin Fallavier, France) and Rnase A (0.1 mg ml^−1^). Nuclei were stained with 300 *μ*l of ice-cold staining solution (0.4 mg ml^−1^ propidium iodide (Sigma, Saint Quentin Fallavier, France), 1.16 mg ml^−1^ spermin and analysed by flow cytometry. All solutions were prepared in a stock solution containing 3.4 mM trisodium citrate, 0.1% (v v^−1^) Nonidet P-40, 1.5 mM spermine, 0.5 mM tribase, pH 7.6. The pre-G1 peak corresponded to apoptotic cells.

## RESULTS

### Effects of incubation of MDA-MB231 cancer cells with monocytes on MMPs secretion

**Figure 1:**
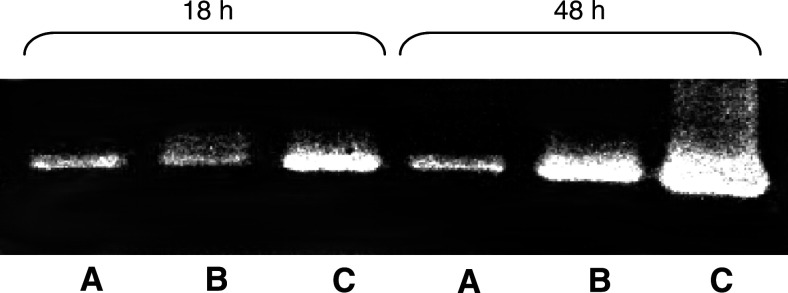
Effect of 18- and 48-h incubation of MDA-MB231 with monocytes on MMP-9 section: zymography study. (**A**) MDA-MB231 alone, (**B**) monocytes alone, (**C**) MDA-MB231 incubated with monocytes.

As shown in Figure 1

, the incubation of MDA-MB231 cancer cells with monocytes induced a strong increase in the band of molecular weight corresponding to MMP-9, which was demonstrated at both 18 and 48 h of incubation.

### Effects of incubation of MDA-MB231 cancer cells with monocytes on membrane expression of invasiveness factors: u-PAR/u-PA system and TF and on u-PA secreted in the conditioned medium

**Figure 2:**
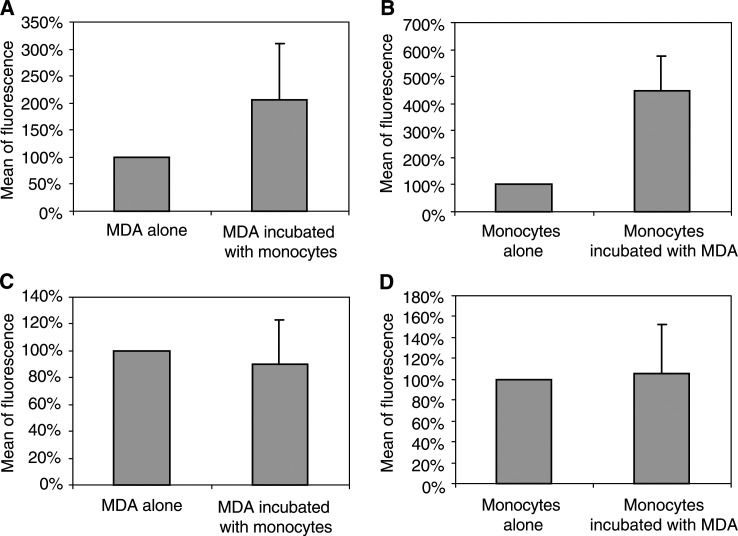
Effect of incubation of MDA-MB231 with monocytes on u-PA and u-PAR expression on each cell type. The u-PA and u-PAR expression was calculated as the percentage of fluorescence as compared with MDA-MB231 and monocytes alone (*n*=4): (**A**) measurement of u-PA associated to MDA-MB231 cancer cells, (**B**) measurement of u-PA associated to monocytes, (**C**) measurement of u-PAR associated to MDA-MB231 cancer cells, (**D**) measurement of u-PAR associated to monocytes.

Membrane expression of u-PA was spontaneously high on MDA-MB231 cancer cells incubated alone and was low on monocytes incubated alone. After incubation of MDA-MB231 cancer cells with monocytes, the u-PA associated with the cell membrane increased in both monocytes and MDA-MB231 cancer cells, as shown in Figure 2

**Figure 3:**
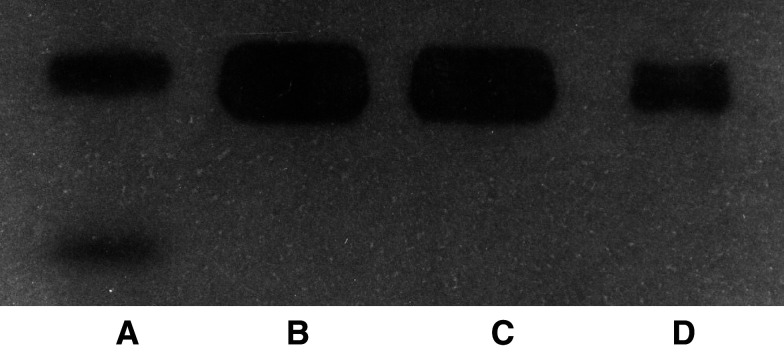
Effect of 48-h incubation of MDA-MB231 with monocytes on u-PA secretion in the conditioned medium. The conditioned medium was submitted to zymography in comparison with purified u-PA: (**A**) purified u-PA, (**B**) supernatant of MDA-MB231 alone, (**C**) supernatant of MDA-MB231 incubated with monocytes, (**D**) supernatant of monocytes alone. Urokinase is visualised as a transparent lysis areas of the gel after 36 h. Two lysis area were observed with purified u-PA, one at 55 kDa molecular weight and a minor one corresponding to the low molecular weight u-PA at 35 kDa.

. However, the incubation of monocytes with MDA-MB231 cancer cells did not modify membrane-associated u-PAR expression either on MDA-MB231 cancer cells or on monocytes (Figure 2). In the conditioned medium, the secretion of u-PA analysed by zymography was not modified when MDA-MB 231 cells were incubated for 48 h with monocytes (Figure 3

).

**Figure 4:**
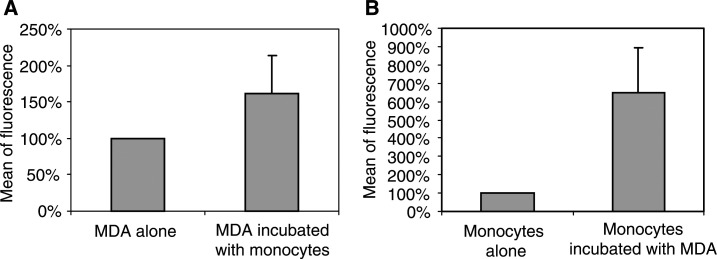
Effect of incubation of MDA-MB231 with monocytes on TF expression on each cell type. The TF expression was calculated as the percentage of fluorescence as compared with MDA-MB231 and monocytes alone (*n*=4): (**A**) measurement of u-PA associated to MDA-MB231 cancer cells, (**B**) measurement of u-PA associated to monocytes.

The TF expressed on membrane was spontaneously high on MDA-MB231 cancer cells. Furthermore, it increased on both MDA-MB231 cancer cells and monocytes after incubating these cells together (Figure 4

).

### Effects of incubation of MDA-MB231 cancer cells and monocytes on OSM, TNF alpha and PAI-1 secretion in supernatants

OSM and TNF alpha were not found in the supernatants of MDA-MB231 cancer cells cultured alone.

The incubation of MDA-MB231 cancer cells with monocytes was responsible for a decreased secretion (61%) of OSM when compared with secretion of monocytes incubated alone.

Also, the incubation of MDA-MB231 cancer cells with monocytes resulted in a dramatic increase in TNF alpha in supernatants (18 000%) as compared with secretion by monocytes incubated alone.

Incubation of MDA-MB231 cancer cells with monocytes induced a mild increase in PAI-1 secretion (135% in comparison to the sum of PAI-1 secreted by MDA-MB231 alone + that secreted by monocytes alone).

These results are shown in Table 1

**Table 1:** Effect of incubation of MDA-MB231 with monocytes on cytokine secretion in supernatants

	**Monocytes alone**	**MDA-MB231 alone**	**Incubation of monocytes with MDA-MB231**
OSM	383±36	0	233±38
TNF alpha	34±33	0	6140±1018
PAI-1	36±2	1064±245	1484±378

*n*=4. MDA-MB 231 cells were cultured in a 24-well culture plate. When the confluence reached 80%, 0.5 × 10^6^ monocytes were added and incubated for 18 h. Controls were performed with MDA-MB 231 alone and monocytes alone. Then the supernatants were collected and cytokines were determined. Results are shown in pg ml^−1^ units.

.

### TNF alpha-induced apoptosis on MDA-MB231 cancer cells

MDA-MB231 cancer cells were very poorly sensitive to TNF alpha (Table 2

**Table 2:** Effect of TNF alpha on the cell cycle and apoptosis in MDA-MB231 cancer cells

	**72-h incubation of MDA-MB231 with**	
**Phase of cell cycle**	**TNF alpha**	**Control medium**
Pre-G1	24.5%	2.1%
G0/G1	47.1%	57%
S	22.7%	30%
G2/M	5.7%	10.9%

*n*=5. TNF alpha-induced apoptosis of MDA-MB231 cancer cells was studied after incubation of cancer cells with TNF alpha at a concentration of 50 ng ml^−1^. The pre-G1 peak is characteristic of apoptotic cells.

). When MDA-MB231 cancer cells were incubated with 50 ng ml^−1^ TNF alpha, less than 25% of cancer cells entered apoptosis (pre-G1 phase) only after a 72-h incubation (Table 2).

## DISCUSSION

The role of immune response in cancer progression seems to be possibly dual and remains not well elucidated (Chambers *et al*, 1997; Jonjic *et al*, 1998; Koukourakis *et al*, 1998; Toi *et al*, 1999; Shimura *et al*, 2000). Indeed, despite the role of monocytes/macrophages in host defence, previous studies have suggested a potential deleterious effect of stroma-localised monocytes/macrophage, in relation with enhanced angiogenesis (Etoh *et al*, 2000; Hashimoto *et al*, 2000; Ueno *et al*, 2000b). In the present study, we investigated the consequences of the interaction between monocytes and aggressive breast-derived MDA-MB231 cancer cells in both the invasiveness capacities of cancer cells and the monocytes functions.

Concerning the role of monocytes in cancer cell invasiveness capacities, this cooperation was analysed on protease secretion reported to be involved in cell migration, that is, u-PA and MMPs. Indeed, evidence has accumulated that invasion and metastasis in solid tumours require the action of tumour-associated proteases, which promote the dissolution of the surrounding tumour matrix and the basement membranes.

We showed that incubation of MDA-MB231 cancer cells with monocytes induced an increased expression of membrane-associated u-PA, on both monocytes and MDA-MB231 cancer cells. It was not associated with modifications of cell-associated u-PAR. In contrast with this increase in membrane-associated u-PA, u-PA secreted in the conditioned medium was not modified when MDA-MB231 cancer cells were incubated with monocytes. This may be explained by the large excess of free u-PAR on cell membrane which bind the secreted u-PA. Interestingly, the increase in membrane-associated u-PA was not observed when monocytes were incubated with the less aggressive breast-derived MCF-7 cancer cells (results not shown).

This increase in u-PA associated to aggressive cancer cells could contribute to the bad prognosis of inflammatory breast cancer. Indeed, a high tumour expression of u-PA is associated with poor prognosis in breast cancer. This is explained by an increased invasiveness of cancer cells as membrane-associated u-PA is involved in cancer cell invasion. Urokinase acts by degradation of pericellular matrix, because of proteolytic cascade focused on cell surface leading to plasmin generation and matrix metalloprotease activation (Andreasen *et al*, 1997) but also by an increased cell migration independent of proteolysis (Silvestri *et al*, 2002). In addition, in relation to plasmin formation, uPA activates latent growth factors and releases growth factors bound to extracellular matrix (Odekon *et al*, 1994; Ribatti *et al*, 1999).

In parallel, it was shown that incubation of MDA-MB231 cancer cells with monocytes increased PAI-1 secretion, which is also a factor contributing to cancer cell invasiveness. Indeed, PAI-1 is a major inhibitor of u-PA, even when associated to cell surface. However, high expression of PAI-1 is paradoxically associated with marked tumour spreading and poor prognosis (Pappot *et al*, 1995; Andreasen *et al*, 1997; Schmitt *et al*, 1997; Look *et al*, 2002).

This pathogenic effect of PAI-1 in cancer aggressiveness was confirmed by the report that cancer invasion and neovascularisation are abolished in PAI-1-deficient mice, and can be restored by local induction of PAI-1 expression (Bajou *et al*, 1998). This increase in PAI-1 allows a critical balance of u-PA associated to its cell surface receptor, as it could represent a key molecule in the rapid attachment/detachment events occurring at the cell leading edge that are required for migration. (Lauffenburger and Horwitz, 1996).

Furthermore, incubation of monocytes with MDA-MB231 cancer cells also induced an increase in u-PA associated to monocytes. This increase could be critical for tumour invasion (Nielsen *et al*, 1996,^2001^), as it has been shown that tumour xenografts grow slowly in u-PA −/− mice (Frandsen *et al*, 2001). The mechanism of u-PA increase on monocytes could be because of a binding of u-PA secreted by MDA-MB231, as it was observed that the amount of u-PA in the conditioned medium of monocytes incubated with MDA-MB231 was higher than that of monocytes alone. It also could be related to an increased production of u-PA by monocytes, secondary to the increased secretion of TNF alpha observed when monocytes were incubated with MDA-MB231. Indeed, TNF alpha stimulates the production of u-PA by monocytes (Gyetko *et al*, 1992).

We also demonstrated that the incubation of monocytes with MDA-MB231 cancer cells induced an increase in MMP-9 secretion. As for u-PA, an increased secretion of MMP-9 is proposed to be involved in cancer invasion and metastasis. Indeed, an increase in MMP-9 secretion has been shown to enhance the invasive capacities of breast cancer cells (Johansson *et al*, 2000). Moreover, MMP-9 was detected more frequently in malignant breast carcinoma than in benign tumour (Rha *et al*, 1997; Hanemaaijer *et al*, 2000). However, the role of MMP-9 is less clear than that of u-PA, as overexpression of MMP-9 in human breast cancer was reported to be a favourable indicator in node-negative patients (Scorilas *et al*, 2001).

After testing incubation of monocytes with MDA-MB231 cancer cells on protease secretion, the cooperation between MDA-MB231 cancer cells and monocytes was analysed on TF expression. The incubation of MDA-MB231 cancer cells with monocytes also induced an increase in cell-associated TF expression in both monocytes and MDA-MB231 cancer cells. TF, an initiator of blood coagulation cascade, is expressed in a wide variety of cancer cells and it is an independent prognostic indicator for overall survival in cancer patients (Ueno *et al*, 2000a). This increase in TF, because of cooperation between cancer cells and monocytes, could contribute to the deleterious effect of monocytes in cancer progression, as it plays an important role in cancer cell invasiveness. Tissue factor is also implicated in both hypercoagulablility in cancer patients (Hu *et al*, 1994) and enhancement of the metastatic potential of cancer cells, probably by induction of angiogenesis (Amirkhosravi *et al*, 1998; Rickles *et al*, 2001). Our result can be an explanation for the observation that TF expressed on monocytes in tumours is particularly abundant in close proximity of invasive tumour cells (Vrana *et al*, 1996).

Concerning the effect of MDA-MB231 cancer cells on monocytes functions relevant for antitumour effect, it was shown that the incubation of monocytes with MDA-MB231 cancer cells resulted in a decrease in OSM secretion, a cytokine which reduces the growth rate of breast cancer cells (Li *et al*, 2001). Therefore, this cooperation induces a reduction of monocyte-dependent host-defence function.

The effect of the incubation of monocytes with MDA-MB231 cancer cells was also tested on TNF alpha, a cytokine secreted by monocytes (Walczak *et al*, 1999). We demonstrated that TNF alpha secretion by monocytes was dramatically increased when incubated with MDA-MB231 cancer cells. However, this increase could not participate in host defence against tumour cells by inducing apoptotic signals, as MDA-MB231 cells are resistant to TNF alpha. Indeed, high concentrations of TNF alpha induced apoptosis for less than 25% of MDA-MB231 cancer cells. This resistance is explained by constitutive activation of NF-KB in MDA-MB231 cancer cells (Denoyelle *et al*, 2001), which prevents TNF alpha-induced apoptosis (Karin and Lin, 2002). Moreover, a correlation between TNF alpha plasma concentration and the severity of the disease in clinical studies has been reported (Sheen-Chen *et al*, 1997). This explains that in cancer patients, instead of mediating cancer cell apoptosis, TNF alpha may be responsible for discomfort symptoms, such as cancer-related fatigue and weightloss (Kurzrock, 2001).

In conclusion, this report underlines the potential role of stroma-localised monocytes in cancer progression. Our results demonstrate the existence of a crosstalk between aggressive breast-derived MDA-MB231 cancer cells and monocytes, which resulted in both the increase in factors involved in cancer cell invasiveness and the decrease in some antitumour effects of monocytes. Moreover, the increase of TNF alpha secretion, instead of inducing cancer cell apoptosis, could produce potential systemic deleterious effects in patients. These findings may be useful to explain the deleterious prognostic significance of stromal monocytes infiltration in cancer.
